# *In silico* analysis of secreted effectorome of the rubber tree pathogen *Rigidoporus microporus* highlights its potential virulence proteins

**DOI:** 10.3389/fmicb.2024.1439454

**Published:** 2024-09-16

**Authors:** Rawit Longsaward, Unchera Viboonjun, Zilan Wen, Fred O. Asiegbu

**Affiliations:** ^1^Department of Plant Pathology, Faculty of Agriculture, Kasetsart University, Bangkok, Thailand; ^2^Department of Plant Science, Faculty of Science, Mahidol University, Bangkok, Thailand; ^3^Forest Pathology Research Laboratory, Department of Forest Sciences, Faculty of Agriculture and Forestry, University of Helsinki, Helsinki, Finland

**Keywords:** effectorome, *Rigidoporus microporus*, rubber tree, virulence, white root rot disease

## Abstract

*Rigidoporus microporus*, the causative agent of the white root rot disease of rubber trees, poses a significant threat to natural rubber production worldwide. Understanding the molecular mechanisms facilitating its pathogenicity would be crucial for developing effective disease management strategies. The pathogen secretes effector proteins, which play pivotal roles in modulating host immune responses and infection. In this study, *in silico* analyses identified 357 putative secreted effector proteins from the *R. microporus* genome. These were then integrated into previous RNA-seq data obtained in response to rubber tree latex exposure. Annotation of putative effectors suggested the abundance of proteins in several families associated with the virulence of *R. microporus*, especially hydrophobin proteins and glycoside hydrolase (GH) proteins. The contribution of secreted effectors to fungal pathogenicity was discussed, particularly in response to rubber tree latex exposure. Some unknown highly expressed effectors were predicted for the protein structures, revealing their similarity to aminopeptidase, ubiquitin ligase, spherulin, and thaumatin protein. This integrative study further elucidates the molecular mechanism of *R. microporus* pathogenesis and offers alternative targets for developing control strategies for managing white root rot disease in rubber plantations.

## 1 Introduction

The white root rot disease (WRD) of rubber tree (*Hevea brasiliensis*), one of the elite crops worldwide, leads to a serious problem especially in the first few years after planting because they can affect water and nutrient uptake, and hence reduce growth and yield of natural rubber (Mohammed et al., 2014). This disease has been suggested to rank high as a major rubber tree catastrophe in many rubber tree planting countries, particularly in Thailand, Malaysia, Indonesia, Sri Lanka, Nigeria, and Cote d’Ivoire, as well as in several other countries (According to CABI compendium).^1^ The disease is caused by the Basidiomycete fungal pathogen *Rigidoporus microporus* (Fr.) Overeem., which can persist on dead root debris for a long time before reaching the living roots and switching to the pathogenic phase (Wattanasilakorn et al., 2012). The symptoms of infected rubber trees manifest in the form of leaf discoloration of leaves (e.g., yellowish brown), early flowering, and fruiting off-season. However, the observation at the initial stage of disease based on these aboveground symptoms requires a trained mind as it was hardly discriminated from symptoms of drought or other abiotic stresses. The only obvious aboveground sign of WRD fungus is the production of the orange basidiocarp with a whitish margin at the lower stem trunk (Omorusi, 2012), which is, unfortunately, considered a severe and late event of the WRD, by which time the whole rubber tree root may already be damaged.

Insights into the molecular responses of rubber trees against *R. microporus* were reported recently by a number of authors. Once the rubber tree root has been infected and colonized by fungus, the systemic molecular responses in the rubber tree can be observed in the roots (Siddiqui et al., 2017), stem (Oghenekaro et al., 2016a; Janket et al., 2022), and leaves (Woraathasin et al., 2017; Longsaward et al., 2023). Several rubber tree defense-related proteins were noticed as the alleviated mechanisms for tolerating WRD disease. Defects in photosynthesis after being infected by *R. microporus* may have contributed to the leaf discoloration and growth retardation of rubber trees.

Understanding the mechanisms of how the *R. microporus* pathogen causes WRD would highly contribute to disease management. Identifying and understanding as many potential virulence factors of the fungal pathogen will help to facilitate the selection of suitable strategies and tools to combat them. Rubber tree roots colonized by *R. microporus* will exhibit observable fungal mycelia that grow firmly attached to the root surface. The woody root then turns creamer and softer due to the activity of cell wall-degrading enzymes released by *R. microporus* to promote its further invasion of plant tissue (Omorusi, 2012). Delignification, a process driven by key enzymes including lignin peroxidases, manganese peroxidases, and laccases, has been reported as one of the fungal virulence that causes root rot symptoms (Paleaz et al., 1995). From the analysis of *de novo* transcriptome, *R. microporus* expresses peroxidases, laccases, aldo-keto reductases, oxidases, and oxidoreductases for depolymerization of rubber lignin during the saprotrophic growth into the rubber wood (Oghenekaro et al., 2016b). Among these, the laccase was proven to have enzyme activity correlated to the pathogenicity of *R. microporus*, the higher virulence isolates showed higher laccase activity (Suwandi, 2007).

Recent breakthrough technologies allow the exploration of the genome sequence from several species, including the WRD fungus *R. microporus*. According to Oghenekaro et al. (2020), the genome analysis in this fungal pathogen revealed a larger number of cellulose-degrading enzymes, than the hemicellulose-and pectin-degrading enzymes. Several protein candidates related to fungal virulence were annotated. Interestingly, there is evidence showing that the rubber tree latex is unable to suppress fungal growth but, conversely, promotes the growth of *R. microporus* with the help of supportive latex degradation genes. This highlights the aggressiveness of *R. microporus* against the host’s basal defenses. Although several small secreted proteins (SSPs) were proposed as effector proteins due to their upregulation when co-culturing with other competitive fungal species (Oghenekaro et al., 2020), none of the studies have systemically identified the putative effector proteins from the *R. microporus* fungus.

Effectors were recently defined as molecules that influence the interaction between organisms in a way that benefits the one who releases or uses them (Todd et al., 2022). This expands the classical definition in plant pathology, where effectors are traditionally referred to as molecules from pathogens that manipulate the host’s cellular functions and structures to support their infection and trigger plant defenses (Kamoun, 2006). According to the Zig-Zag model (Jones and Dangl, 2006), pathogens employ effectors to bypass the basal pattern-triggered immunity (PTI) expressed by the host. Microbial pathogens have developed various effectors as sole strategy to interfere with several molecular functions of their hosts including (i) proteins (Alfano and Collmer, 2004; Stergiopoulos and de Wit, 2009), (ii) small RNAs (Weiberg et al., 2013; Wang et al., 2017), and (iii) secondary metabolites (Friesen et al., 2008; Stergiopoulos et al., 2013; Collemare et al., 2019) especially polyketide derivatives (Hassani et al., 2022). Effectors are crucial to the virulence of pathogens and largely contribute to the pathogenesis in suppressing or avoiding the host immune systems. Fungal effectors can be divided based on their pathogenic functions into biotrophic effectors, which facilitate colonization in the host via suppressing plant immunity (Uhse and Djamei, 2018), and necrotrophic effectors that highly disrupt host cells such as phytotoxins and cell wall-degrading enzymes (Kim et al., 2016; Shao et al., 2021; Samperna et al., 2022). The hemibiotrophic fungi on the other hand could use either biotrophic or necrotrophic effectors as adaptive modes for their lifestyles (Kabbage et al., 2015). Therefore, the information on effectors utilized by particular pathogens and the understanding of their function could benefit the effective disease management of crop plants or forest trees.

Identification of all effectors in an organism, a so-called effectoromics analysis, provides a broader understanding of fungal virulence and lifestyle (Todd et al., 2022). However, the identification of the effectorome is highly challenging from the determination of how effectors should be defined, so it influences the elucidation and identification methods. Regarding effector proteins, the taxonomic distribution of effector proteins is limited and they contain very low or no sequence similarity to other protein families or other organisms. The lack of homology among effectors leads to a fewer number of effector protein families. To date, the effectors can also be defined as the (i) canonical effector proteins that are usually cysteine-rich proteins of less than 300 amino acid residues with the presence of signal peptide, and their genes are highly expressed during the interaction with the host or other competitive microbes, and (ii) the non-canonical effector proteins that implemented the well-known conserved domains and motifs from other known pathogenic effector regardless of the protein size and protein secretion (Todd et al., 2022).

To explore the effectoromic in the WRD fungus, the secreted proteins were *in silico* screened from the total proteins in the *R. microporus* genome (Oghenekaro et al., 2020). Thereafter, the secreted protein candidates were predicted for the effector characteristics in this study, through the Effector P prediction tool and the search for effector-conserved motifs. Several protein families identified as potential effector proteins were compared to the other relative fungal species and also discussed with the available transcriptome dataset of genes expressed in response to latex, a rubber tree basal defense. In addition, some of the key *R. microporus* virulent effectors with low sequence homology were predicted for their structures. The integrative information of *R. microporus*-secreted effectors would accelerate the applications for effective disease management in future, especially the chemical or biofungicide targeting of these virulence factors of WRD fungus. Moreover, the information would benefit in prioritizing effector candidates from *R. microporus* for further characterization in the lab.

## 2 Materials and methods

### 2.1 Effector identification

The fungal proteome, acquired from the JGI genome portal of *Rigidoporus microporus* ED310 v 1.0 (Oghenekaro et al., 2020) (accessed on November 20, 2023),^2^ was used for the *in silico* identification of effector candidates using steps of machine learning-based tools available online. To identify potential fungal effectors, the 10,917 proteins of *R. microporus* were subjected to secreted protein prediction by Signal P 6.0 (Teufel et al., 2022) (accessed on November 21, 2023).^3^ Then, the secreted protein candidates were screened for effector potential by (i) Effector P 3.0 (accessed on November 21, 2023)^4^ (Sperschneider and Dodds, 2022) and (ii) a search for effector-conserved motifs using Find Individual Motif Occurrence (FIMO) v. 5.5.5 (accessed on January 31, 2024)^5^ (Grant et al., 2011). In this study, the predicted apoplastic effectors and cytoplasmic effectors from Effector P as well as the secreted proteins with effector-conserved motifs were considered as effectors of the *R. microporus* fungus.

### 2.2 Domain analysis of secreted proteins

The secreted protein candidates were retrieved for their related information from the MycoCosm database,^6^ i.e., the amino acid sequences, protein ID, transcript ID, domain-associated protein family (PFam), and GO terms. All candidates were re-predicted for the protein domain via HMMscan (accessed on 15 January 2024)^7^ (Potter et al., 2018) in order to resolve those candidates without annotation information from MycoCosm database.

### 2.3 Analysis of secreted protein expression from the transcriptome data

The expression of secreted protein candidates identified here was retrieved from the transcriptomic analysis of *R. microporus* in response to natural latex treatment. The transcriptome data in NCBI BioProject 497786^8^ were previously analyzed for differentially expressed genes (DEGs) by comparing three replicates of pure *R. microporus* culture supplemented with rubber wood to three replicates of pure *R. microporus* culture supplemented with rubber wood or rubber latex (Oghenekaro et al., 2020). The protein candidates in this study were screened from the list of DEGs and their expression data were used to discuss the potential molecular mechanisms of the fungi during interaction with the rubber tree.

### 2.4 Prediction of uncharacterized effectors

The structural-based prediction of highly expressed effectors that were previously considered unknown proteins was carried out through the D-I-TASSER server.^9^ The server predicted the secondary-and 3D structures, ligand binding sites, enzyme activity, and GO terms from the template protein in the PDB database. The protein structure was visualized by UCSF Chimera v. 1.15.

## 3 Results

**Figure 1:**
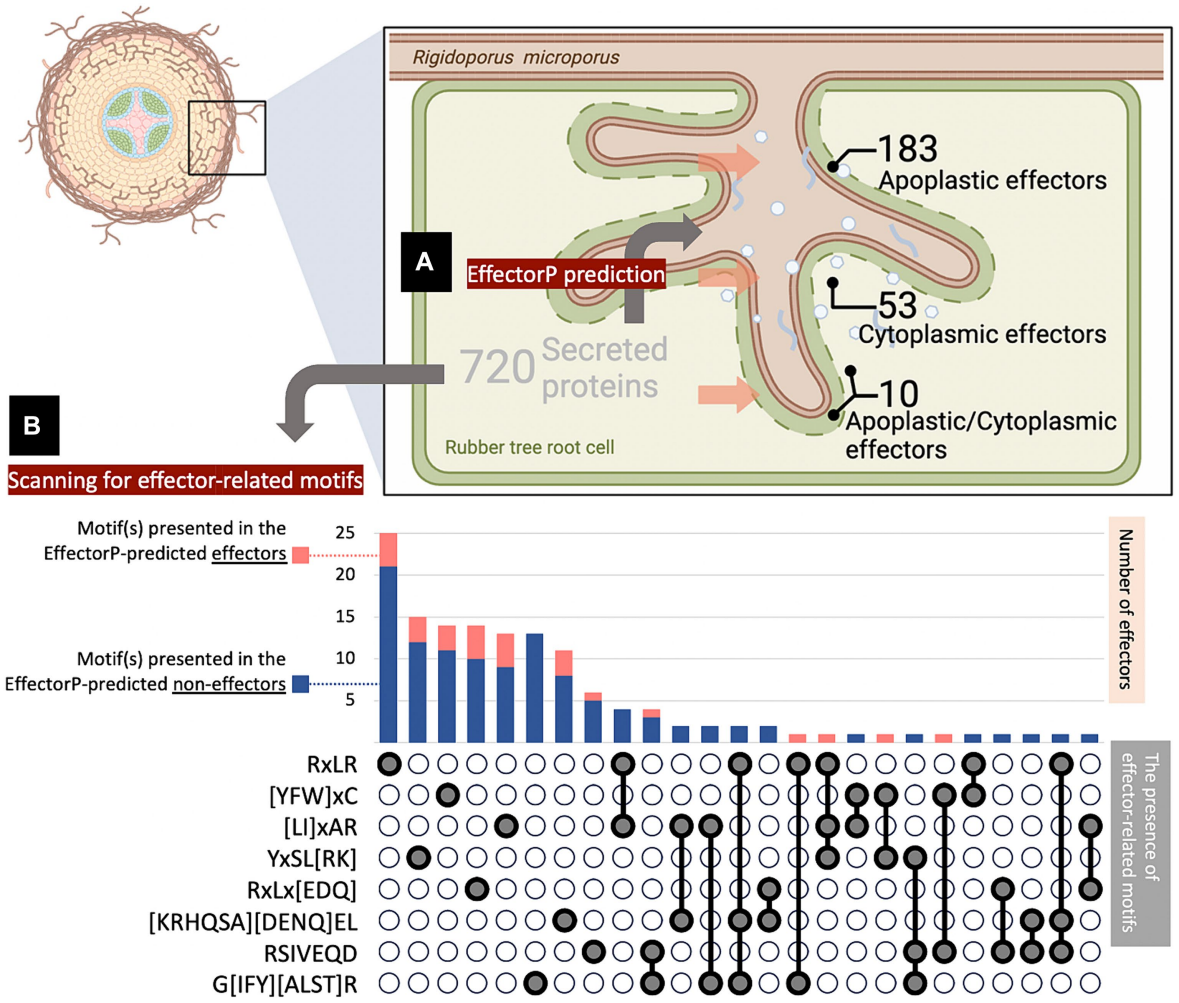
Number of effector candidates identified *in silico* from the *Rigidoporus microporus* fungus. A total of 720 proteins were predicted as secreted proteins, of which 246 effectors were predicted by the Effector P tool, and another 111 effectors were predicted by FIMO analysis of effector-conserved motifs. **(A)** Effector P predicted 183 apoplastic effectors, 53 cytoplasmic effectors, 10 apoplastic/cytoplasmic effectors, and 474 non-effectors. The penetration of fungus into the root was designed by BioRender. **(B)** The number of effectors with conserved motifs was investigated by FIMO analysis. The presence of one-, two-, or three-motifs on individual effectors was indicated in gray below the stacked chart, which shows the number of effector proteins predicted as effectors by Effector P (pink) and those overlooked by Effector P (navy). Each of the selected motifs was searched across the 720 secreted protein sequences identified in this study, using FIMO analysis with an *E*-value cut-off of 0.0001 (Supplementary Table S4).

In this study, the candidate proteins potentially acting as effectors of the pathogenic fungus *R. microporus* were analyzed using machine learning-based tools available online. A total of 10,917 unique proteins reported in the *R. microporus* genome were assessed for the secretory feature by Signal P v. 6.0, revealing 720 proteins with high-potential signal peptides (Supplementary Table S1). These secreted proteins were then scrutinized for their effector characteristics by Effector P 3.0 (Supplementary Table S1). Among the 720 secreted proteins, Effector P predicted 183 as apoplastic effectors, 53 as cytoplasmic effectors, 10 proteins as both apoplastic and cytoplasmic effector features, while the remaining 474 candidates were categorized as non-effector-secreted proteins (Supplementary Table S2; Figure 1A).

To expand the effector identification, well-known motifs conserved in fungal effectors were focused and used as criteria to determine the effector-likeliness of secreted proteins from *R. microporus*. The presence of effector-related motifs in the candidate sequences was addressed using the FIMO analysis tool. Consequently, the analysis identified additional effectors containing these selected motifs (Figure 1B; Supplementary Table S4). This included 27 effector proteins predicted by Effector P and another 111 additional proteins that were overlooked by Effector P prediction (Supplementary Table S3). As a result, a total of 357 effector proteins have been identified as the effectorome of *R. microporus* in this study comprising 246 effectors predicted by Effector P and 111 effectors containing conserved fungal effector motifs (Figure 1).

**Figure 2:**
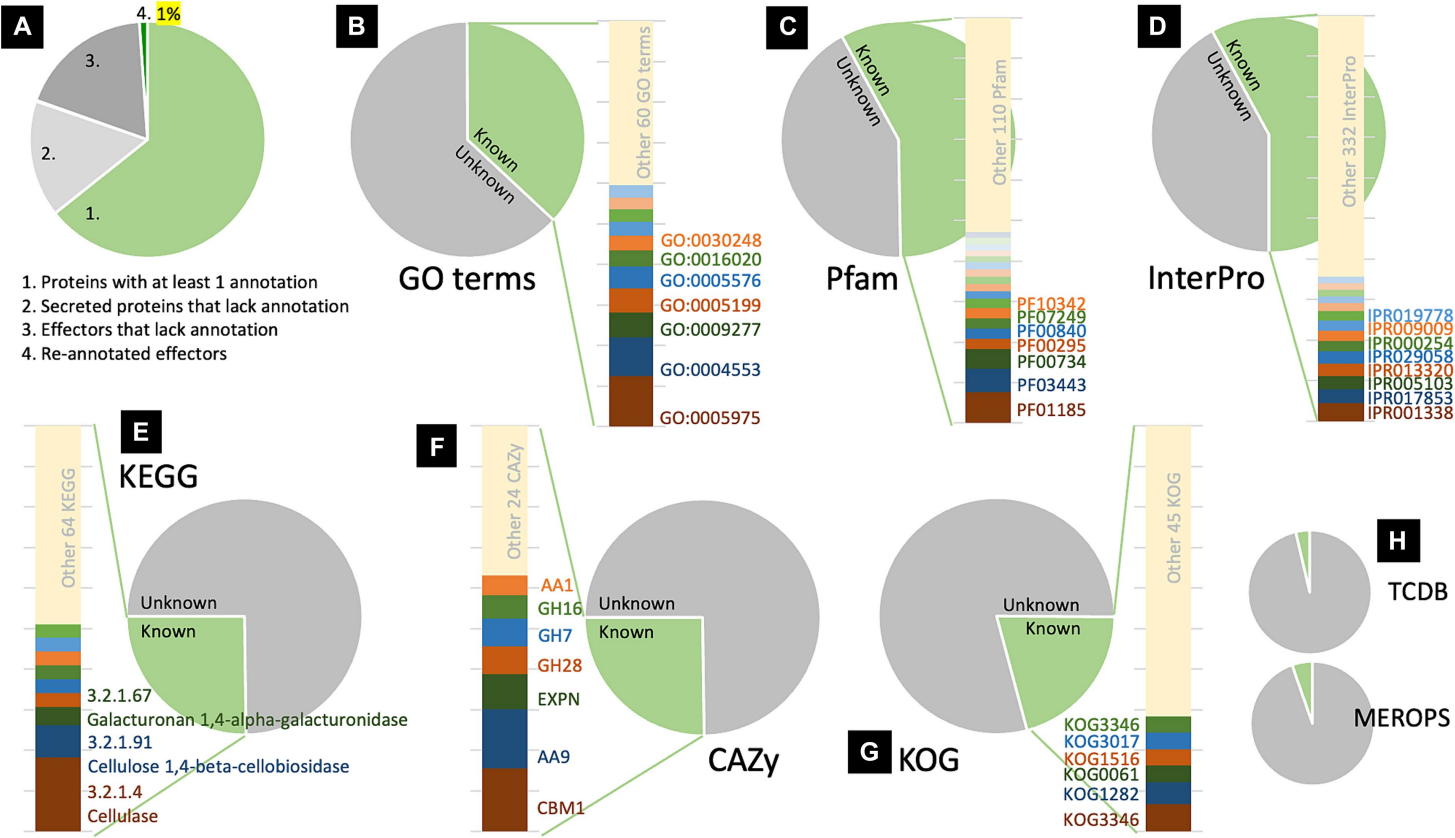
Analysis of protein information revealed the proportion of annotated and unannotated effector proteins of *Rigidoporus microporus* identified in this study. Approximately 64% of secreted proteins were annotated to at least one annotation based on the dataset of the *R. microporus* genome in the MycoCosm database **(A)**. The effector proteins are analyzed for their GO terms **(B)**, Pfam accessions **(C)**, InterPro entries **(D)**, KEGG **(E)**, CAZy **(F)**, KOG **(G)**, as well as TCDB and MEROPS **(H)**. The proportion of each known annotation was plotted as a stacked chart.

The information associated with the secreted proteins and effectors identified in this study was obtained from the fungal genomic resource of *R. microporus*. The information includes annotation of Gene Ontology (GO) terms, Classification of Protein Families by Pfam and InterPro entry, Kyoto Encyclopedia of Genes and Genomes (KEGG), Carbohydrate-Active Enzymes (CAZy), Conserved Protein Domain Family (KOG), Transporter Classification Database (TCDB), and Peptidase database (MEROPS) (Supplementary Tables S2, S3). Among the 720 secreted proteins, 463 have been annotated with at least one of the aforementioned categories (64.31%), while 257 remain unannotated (35.69%), of which 141 were identified as effectors (Figure 2A). Consequently, further analysis was conducted on the protein re-annotation of these effectors and other secreted proteins using hmmscan for protein domain identification (Supplementary Tables S2, S3). This process improved the annotation, matching 8 previously unannotated effectors and 1 other unannotated secreted protein to Pfam, accounting for 1% of the secretome (Figure 2A).

To investigate the annotation profiles of *R. microporus* effectorome, the information of 357 effectors was thoroughly analyzed across each of the 8 annotation tools (Figures 2B–H). The datasets from Pfam and InterPro were more informative for the *R. microporus* effectorome than GO terms and other annotations (Figure 2). Figures 2C,D showed that 42% of the effector proteins were associated with known protein families. The results revealed that a total of 128 PFam domains and 346 InterPro entries are associated with 206 and 207 effectors, respectively. However, 151 effectors still lack the matched Pfam domain annotations by hmmscan. Among the annotated domains, the 9 most frequently encountered Pfam domains in *R. microporus* effectors are listed (Table 1), highlighting the prevalence of domains such as fungal hydrophobin domain (PF01185), auxiliary activity family 9 domain (PF03443), fungal cellulose binding domain (PF00734), and others.

**Table 1:** The top 9 most abundant protein domains presented in *R. microporus* effector proteins.

Pfam	PFam description	Number of effectors		
		Total	Single known domain	With other known domain
PF01185	Fungal Hydrophobin	21	20	1
PF03443	Auxiliary Activity family 9 (former Glycosyl Hydrolases family 61)	16	15	1
PF00734	Fungal Cellulose-Binding domain	13	4	9
PF00295	Glycosyl Hydrolases family 28	7	7	
PF00840	Glycosyl Hydrolases family 7	7	4	3
PF07249	Cerato-platanin	7	7	
PF10342	Kre9/KNH-like N-terminal Ig-like domain	7	7	
PF00722	Glycosyl Hydrolases family 16	6	6	
PF01105	Emp24/gp25L/p24 family/GOLD	5	5	

The transcriptomic data of *R. microporus* change upon the treatment of rubber tree latex, and based on the experiment by Oghenekaro et al. (2020), it was utilized to analyze effector candidates potentially involved in the rubber tree-*R. microporus* interaction. Among the 357 identified effectors, 319 were differentially expressed while 38 showed no significant changes upon latex treatment (Supplementary Tables S2, S3). Table 2 illustrates the significant differential expression rank of effector candidates with some abundant domains outlined in Table 1 and Figure 2. Using the threshold at a 1.5-fold difference, the 17 candidates with significantly lower expression levels and the 14 with significantly higher expression levels are listed. The results highlight the prominent roles of effectors containing fungal hydrophobin domains, which exhibited robust responses of *R. microporus* to rubber tree latex.

**Table 2:** List of the differentially expressed *Rigidoporus microporus* effectors that significantly change (more than 1.5 Log2Fold) following the treatment with rubber tree latex with the low expression level (A) and the high expression level (B) based on the transcriptome data reported by Oghenekaro et al. (2020).

(A) The *R. microporus* effectors with significant lower expression levels following treatment with rubber tree latex							
Rank	Log_2_ fold change	Protein ID	Annotation	InterPro	Pfam	CAZy	Conserved motif
1	−3.675	862870	Thaumatin family	IPR001938	PF00314	GH152	–
2	−3.409	943343	Fungal Hydrophobin	IPR019778 IPR001338	PF01185	–	–
3	−3.299	290678	Fungal Hydrophobin	IPR019778 IPR001338	PF01185	–	–
4	−2.720	864959	Fungal Hydrophobin	IPR019778 IPR001338	PF01185	–	–
5	−2.644	844792	Fungal Hydrophobin	IPR019778 IPR001338	PF01185	–	–
6	−2.635	948486	Egh16-like Virulence Factor	IPR021476	PF11327	–	–
7	−2.474	1013727	Unknown	–	–	–	–
8	−2.029	835459	Auxiliary Activity family 9	IPR005103	PF03443	AA9	–
9	−1.874	1009326	Fungal Cellulose Binding domain	–	PF00734	–	–
10	−1.697	826741	Fungal Hydrophobin	IPR019778 IPR001338	PF01185	–	–
11	−1.593	924601	Thaumatin family	IPR001938	PF00314	GH152	–
12	−1.583	945455	Unknown	–	–	–	[YFW]xC
13	−1.551	892384	Egh16-like Virulence Factor	IPR021476	PF11327	–	–
14	−1.546	949558	Unknown	–	–	–	–
15	−1.537	836667	Glycoside Hydrolase Family 27	IPR000111 IPR017853 IPR002241	PF02065	GH27	RxLR, [YFW]xC
16	−1.526	832344	Glycosyl Hydrolase Family 28	IPR011050 IPR000743	PF00295	GH28	G[IFY][ALST]R, RSIVEQD
17	−1.525	827302	Fungalysin	IPR011096 IPR001842	PF07504 PF02128	–	G[IFY][ALST]R, RSIVEQD

(B) The *R. microporus* effectors with significant higher expression levels following treatment with rubber tree latex							
Rank	Log_2_ fold change	Protein ID	Annotation	InterPro	Pfam	CAZy	Conserved motif
1	3.821	872163	Auxiliary Activity family 9	IPR005103	PF03443	AA9	–
2	2.976	882769	Unknown	–	–	–	–
3	2.953	872712	Unknown	–	–	–	–
4	2.912	921543	Aspartyl Protease	IPR001461 IPR021109	PF00026	–	G[IFY][ALST]R
5	2.783	863046	Pro-kumamolisin	IPR015366 IPR030400 IPR000209	PF09286	–	[KRHQSA][DENQ]EL
6	2.565	884303	Fungal Hydrophobin + Collagen triple helix repeat	IPR008160 IPR001338	PF01185 PF01391	–	–
7	2.148	832388	Fungal Hydrophobin	IPR019778 IPR001338	PF01185	–	–
8	2.092	467301	Unknown	–	–	–	G[IFY][ALST]R
9	2.055	942071	Unknown	–	–	–	–
10	1.939	941140	Unknown	–	–	–	–
11	1.809	832495	Fungal Hydrophobin	IPR019778 IPR001338	PF01185	–	–
12	1.619	89931	Unknown	–	–	–	–
13	1.602	885660	Cysteine-rich secretory protein family	IPR018244 IPR001283 IPR014044	PF00188	–	–
14	1.599	948612	Unknown	–	–	–	–

**Figure 3:**
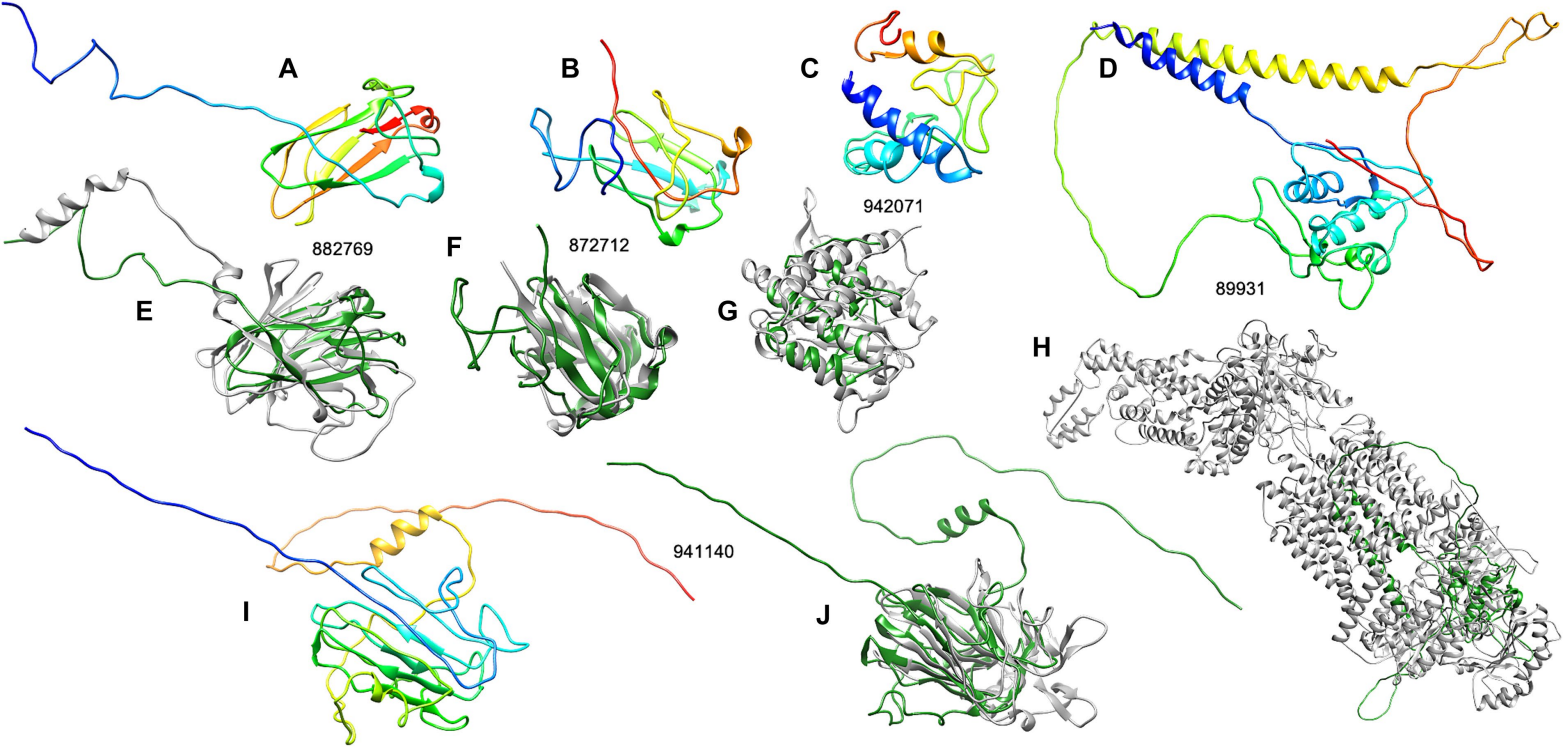
The structure analysis of five unknown *R. microporus* effectors whose expression was highly induced upon the interaction with rubber tree latex. The 3D structures of each candidate effector were predicted by the D-I-TASSER server **(A–C,I)** and compared with the most relevant template structure **(E–H,J)** including effector protein 882769 **(A)** compared with the PDB: 8PD4A **(E)**, effector protein 872712 **(B)** compared with the PDB: 1HDFA **(F)**, effector protein 942071 **(C)** compared with the PDB: 5JQKA2 **(G)**, effector protein 89931 **(D)** compared with the PDB: 7TBWA **(H)**, and effector protein 941140 **(I)** compared with the PDB: 2AHNA **(J)**. The color range of the 3D structures represents the N-terminal (blue) to the C-terminal (red) of each candidate effector **(A–C,I)**. The structural comparison showed the similarity of effector (green) and the PDB template (gray) **(E–H,J)**.

The results showed 7 of the 14 highest expressed effectors with no annotation to any types of proteins, including the proteins 882769, 872712, 467301, 942071, 941140, 89931, and 948612 (Table 2B). To understand more about how some of these unknown latex-responsive effectors could contribute to the resilience of the pathogen to plant defense, the protein structural-based prediction was then performed by the D-I-TASSER server (Figure 3). Additional information on five previously uncharacterized effector proteins, including 882769, 872712, 942071, 941140, and 89931, was uncovered (Table 3).

**Table 3:** The structurally related proteins in PDB that were selected as templates for the prediction of unknown *R. microporus* effector protein structures by the D-I-TASSER server.

Effector protein	Threading template by sequence	Template protein name [Species]	Normalized Z-score of sequence threading	Threading program used by D-I-TASSER	The model confidence of the top final model	Structural analogous protein of the top final model	Structural-analogous protein name [Species]	TM-score of model structure vs. analog structure
882769	8pd4A	E3 ubiquitin-protein ligase TRIM58 [*Homo sapiens*]	1.00	DEthreader	0.68	8pd4A	E3 ubiquitin-protein ligase TRIM58 [*Homo sapiens*]	0.51
	8k8eA	Nicastrin [*Homo sapiens*]	1.00	DEthreader				
872712	5z6dA1	Abundant perithecial protein (APP) [*Neurospora crassa*]	1.83	SPARKS-K	0.74	1hdfA	Spherulin 3A [*Physarum polycephalum*]	0.58
	1hdfA	Spherulin 3A [*Physarum polycephalum*]	1.83	HHsearch				
942071	7m7eB	6-Deoxyerythronolide B synthase (DEBS) [*Saccharopolyspora erythraea*]	1.00	DEthreader	0.45	5jqkA2	Aminopeptidase [*Plasmodium falciparum*]	0.50
	5dztA	Class II Lanthipeptide synthase CylM [*Enterococcus faecalis*]	1.00	DEthreader				
	6m5aA	Beta-L-arabinobiosidase [*Bifidobacterium longum*]	1.00	DEthreader				
941140	2ahnA	Thaumatin-like Pru av. 2 allergen [*Prunus avium*]	4.30	HHsearch	0.63	2ahnA	Thaumatin-like Pru av. 2 allergen [*Prunus avium*]	0.44
89931	2kluA	T-cell surface glycoprotein CD4 [*Homo sapiens*]	2.05	HHsearch	0.66	7tbwA	ATP-binding cassette, subfamily A (ABC1) [*Homo sapiens*]	0.42

The D-I-TASSER server used 10 threading programs to search against the PDB database for the structurally related structures to the query effector sequence and the template structure with a reliable normalized Z-score (> 1.00) were listed here. The similarity between the effector and template was evaluated as the TM-score (0–1).

The three effectors, including proteins 882769, 872712, and 942071, are structurally related to the E3 ubiquitin ligase protein, spherulin protein, and aminopeptidase protein, respectively, with reliable similarity scores (Table 3). The visualization of the structural comparison showed the likeliness of the known protein despite their low sequence similarities to any of the associated PFam accessions (Figures 3E–G). Meanwhile, the central domain of protein 941140 showed a structural similarity to the thaumatin-like allergen of sweet cherry (Table 3), but the structure of this effector exhibited the extended N-and C-terminal further than the allergen structure (Figure 3J). The predicted structure of effector 89931 was highly unique (Figure 3D) and, however, distinct from the closest structural template ATP-binding cassette structure (Figure 3H).

## 4 Discussion

Effector proteins of phytopathogenic fungi play vital roles during invasive growth, especially those secreted proteins with enzyme activity capable of altering host structural barriers and interfering with cellular immune components. Analyses of bioinformatic data by the currently developed tools, especially the machine-learning-based algorithms, have allowed the more feasible effectoromic studies extended from the wet lab in profiling the secreted proteomics and metabolomics of fungal pathogens. The identification could be done computationally by analyzing the conserved sequences among previously proven effectors and then predicting the likelihood of query sequences from the genome (Todd et al., 2022). The knowledge from the effectoromics study has been proposed to lead to alternative disease controls by targeting the virulent factors of particular pathogens (Vleeshouwers et al., 2011; Lanver et al., 2017; Lin et al., 2023). Although the molecular basis of WRD-rubber tree interactions has been studied (Oghenekaro et al., 2016a; Oghenekaro et al., 2016b; Oghenekaro et al., 2020; Janket et al., 2022; Longsaward et al., 2023), none of the previous research focused on the effectorome in *R. microporus* fungus.

The effector proteins are predicted and characterized using the Signal P server (Teufel et al., 2022) and the Effector P server (Sperschneider and Dodds, 2022). Employing the Signal P and Effector P predictions, the effectors of *R. microporus* were *in silico* analyzed, which led to the identification of 246 protein candidates as secreted effector proteins (Figure 1). The secreted effectors from plant pathogenic fungi are proposed to reach the extracellular space and then target the host components in the apoplast, or enter the cytoplasm to combat the intracellular defense of the host. Using the criteria of Rocafort et al. (2020), predicted by the Effector P server, *R. microporus* effectors could mainly be categorized into 183 apoplastic, 53 cytoplasmic, and 10 apoplastic/cytoplasmic effectors.

Effector-related motifs such as a well-known RxLR motif, which is renowned for mediating the entry of oomycetes and fungi into host cells (Kale et al., 2010; Sonah et al., 2016; Whisson et al., 2016), and the effector-conserved Y/F/WxC motif (Godfrey et al., 2010; Seong and Krasileva, 2023; Cao et al., 2023) were documented. Effector-related motifs were sometimes also used for the identification of non-canonical effectors. Noncanonical effectors are effectors with contrary characteristics such as containing more than 400 amino acids (Bohnert et al., 2004), few cysteine residues (Gout et al., 2006), and no signal peptide (Liu et al., 2014). These are normally submerged under the *in silico* identification of effector based on the canonical characters. In *Pucinia graminis*, Zhao et al. (2020) searched the effector-related motifs to identify the novel effector candidates and found 719 RxLR effectors, 138 Y/F/WxC effectors, and other groups of effectors related to other specific motifs. The RxLR motif-containing effectors of *Phytophthora kernoviae* have been identified and shown to contribute to pathogenicity (Wang et al., 2021). In this study, several effector-related motifs were identified in the sequence of secreted *R. microporus* proteins, including those predicted by Effector P through *in silico* analysis. An additional 111 effectors were further identified from the *R. microporus* secretome that had been overlooked by Effector P prediction. The most frequently found motif is the RxLR motif, which is presented in 33 effector candidates (Figure 1B). This includes 25 effectors containing only the RxLR effector-conserved motif and 8 effectors with the combination of RxLR and other effector-conserved motif(s). Among them, 1 effector in Emp24/gp25L/p24 family/GOLD family (PF01105), and another effector protein ID 915929 are contained RxLR. The later effector protein ID 915929 consists of the domain of tetratricopeptide-like repeats (PF13414) and DNAJ domain (PF00226), and it interestingly has 3 effector-related motifs including RxLR, [LI]xAR, and YxSL[RK] motifs (Supplementary Table S2). However, the relationship between these identified motifs and the effector functions in *R. microporus* pathogenicity still requires further experimental investigation. Future studies should prioritize candidate effectors with conserved motif(s) to better understand their roles in pathogenicity.

The localization where the effector protein from *R. microporus* was predicted to function (Figure 1A) may somehow be related to the role of the effector during the pathogenicity of WRD. Supplementary Table S2 in this study notes the identified *R. microporus* effectors with the characteristics of apoplastic effectors, cytoplasmic effectors, and versatile ones. Regardless of the uncharacterized effectors that are unable to be categorized into any protein family, several annotated protein families of *R. microporus* effectors showed specificity to the localization. The key cytoplasmic effectors are those effector proteins in families PF01105 (*n* = 5), PF00085 (*n* = 3), etc. (Supplementary Table S2). The five cytoplasmic effectors with the domain of PF01105 are in the Emp24/gp25L/p24 family/GOLD family, which function for the secretion via the Golgi apparatus (Anantharaman and Aravind, 2002). The GOLD domain in the Pb257 effector protein was recently proven to be crucial for the virulence of *Plasmodiophora brassicae* in killing the plant cells and causing the clubroot symptom (Yang et al., 2024). Moreover, the three cytoplasmic effectors with thioredoxin domain (PF00085) are noted. In a soilborne fungal pathogen *Verticillium dahlia*, thioredoxins were suggested as a key secreted virulent factor contributing to the scavenging of defensive ROS from host plants (Tian et al., 2023). However, most cytoplasmic effectors in *R. microporus* showed decreased expression upon latex treatments (Supplementary Table S2). The effector protein ID 942071 is the only uncharacterized cytoplasmic effector that showed higher transcription (2.055-fold) in response to latex treatment (Table 2B; Supplementary Table S2), and it was predicted to be structurally closest to the aminopeptidase protein (Table 3; Figure 3G). The aminopeptidase-like effector from *R. microporus* may be crucial for cleaving the peptide from rubber tree latex and facilitating fungal pathogen growth upon latex treatment, as experimentally proven by Oghenekaro et al. (2020). This resembles the virulent peptidases from several plant pathogenic fungi such as peptidases from *Zymoseptoria tritici* and relatives (Krishnan et al., 2018), and two secreted peptidases from *Fusarium oxysporum*, FoAPY1 (Qian et al., 2022) and FolAsp (Wang et al., 2023).

Apoplastic effectors secreted by fungal pathogens are crucial for the pathogenicity of particular diseases as they can be detected by the host apoplastic immune response, and consequently trigger the disease symptoms, and/or, can bypass the plant defense by deactivating the protective molecules in apoplast (Chen et al., 2023). A total of 183 secreted proteins in the *R. microporus* genome were predicted to be apoplastic effectors (Figure 1A; Supplementary Table S2), although most of them remain uncharacterized and unknown. The annotated apoplastic effectors in WRD fungus largely consist of the abundant protein families listed in Table 1, except the earlier mentioned cytoplasmic effectors in family PF01105.

It is worth highlighting the effector proteins in the hydrophobin family (PF01185) as one of the major effectors of *R. microporus* (*n* = 21) because of their abundance in the genome (*n* = 28) and their differential expression in response to the latex application (Table 2). Hydrophobin proteins are important proteins located on the surface of fungi, contributing not only to the fungal pathogenicity but also to the growth and formation of the fruiting body (Karlsson et al., 2007; Mgbeahuruike et al., 2012). In the pathogenicity of filamentous fungal pathogens, hydrophobins are reported to be involved in appressorium formation and mediated attachment to the host surface and subsequent infection (Linder et al., 2005). In the biological control activity, hydrophobins were found to be the major expressed proteins in *Trichoderma atroviride* during the interaction with plants and with the *Rhizoctonia solani* pathogen (Guzman-Guzman et al., 2017). These suggest their importance in the virulence of fungi. In the WRD pathosystem, Oghenekaro et al. (2016b) explored the transcription of *R. microporus* genes during the invasion of rubber tree wood. Over 2.5-fold higher abundance of two transcripts encoding hydrophobins (CL996.Contig2 and Unigene3334), as well as the 145-fold lower abundance of another hydrophobin (CL2382.Contig2) were observed in *R. microporus* when growing on the rubber wood-supplemented media. Accordingly, the integration of the identified effectors with transcriptomic data here revealed the dual pattern of hydrophobin expression upon the response to rubber tree latex (Table 2). In total, four *R. microporus* hydrophobins (Table 2A) showed sharp downregulation lower than 2-fold compared to the latex-free control. Meanwhile, a triple of hydrophobin proteins (Table 2B) showed high upregulation. Interestingly, one of the top upregulated effectors is the hydrophobin protein with collagen triple helix repeat domain (PF01391) whose protein ID is 884303 and its increased expression was 2.6 fold upon latex treatment (Table 2B). These datasets suggest some hydrophobins as *R. microporus* apoplastic effectors that are being suppressed by rubber tree latex, and some redundant hydrophobins as another set of effectors to compensate for the virulence of *R. microporus*.

**Figure 4:**
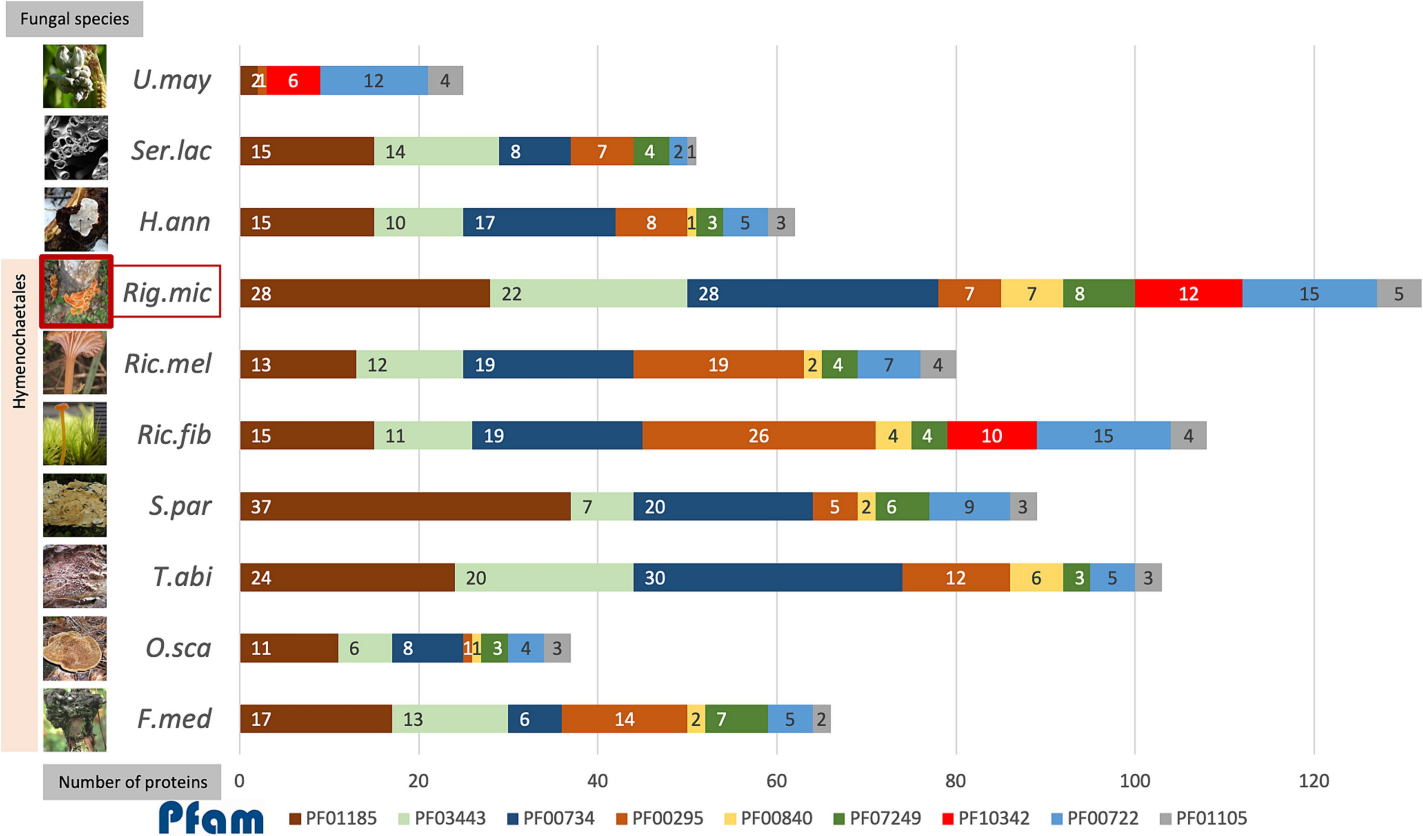
The accumulative bar chart showed the number of proteins encoding each of the top 9 protein families identified as effectors in *R. microporus* (*Rig.mic*) compared to other representative species in the order Hymenochaetales, including *Fomitiporia mediterranea* (*F.med*), *Onnia scaura* (*O.sca*), *Trichaptum abietinum* (*T.abi*), *Schizopora paradoxa* (*S.par*), *Rickenella fibula* (*Ric.fib*), and *Rickenella mellea* (*Ric.mel*), as well as the white rot pathogen *Heterobasidion annosum* (*H.ann*), brown rot fungus *Serpula lacrymans* (*Ser.lac*), and the biotrophic pathogen *Ustilago maydis* (*U.may*). The data on the number of proteins in the fungal genome belonging to each Pfam was collected from the Multigene Clusters (MCL) of the *R. microporus* genome in the MycoCosm database (based on the running Rigmic1-comparative.1858) and of the 3 other pathogens *H.ann*, *Ser.lac*, and *U.may* individually. The fungal images were retrieved from the description of each fungus in the MycoCosm database.

The secreted fungal effectors seem to largely target the host structural barrier, especially the cell-wall-related carbohydrate macromolecules. The high number of *R. microporus* effectors containing the fungal cellulose binding domain (PF00734) are identified in this study as another group of apoplastic effectors (Supplementary Table S2) as well as the effectors presenting conserved motifs (Supplementary Table S3). These effectors are often associated with glycoside hydrolases (GH) domains and have been annotated within the CAZy database, which categorizes carbohydrate-hydrolyzing enzymes. Several studies on fungal effectorome analysis have utilized the CAZy annotation as a classification tool for effectors. For instance, it has been applied in studies of mycorrhizal populations (Kohler et al., 2015; Martin et al., 2016), endophytic fungi in the genus *Hymenoscyphus* (Rafiqi et al., 2023), and the palm dieback pathogen (Rafiqi et al., 2022), where their effectors comprised a significant portion of GHs and auxiliary activity (AA) enzymes. In this study, analysis of the CAZy information for *R. microporus* effectors revealed CBM, GHs, and AAs, as some of the most frequently observed activities (Figure 1F). GH is a large protein superfamily consisting of several classes of GH protein families that exhibit enzymatic cleavage on specific glycosidic bonds of various polysaccharides (Shrivasrava, 2020). Classes of GH proteins that have been considered are mostly cell-wall-degrading enzymes that are extensively mentioned to be important proteins that contribute to the pathogenicity of fungal pathogens in disrupting the host cell structure (Lopez et al., 2018; Bradley et al., 2022). Here, several proteins belonging to the formerly-named GH class 61 (GH61), which is now designated auxiliary activity 9 (AA9) protein (PF03443), were identified as apoplastic effectors of *R. microporus* (Table 1). The expression profile of AA9 proteins is mostly shown to be suppressed upon latex treatment (Supplementary Table S2), except for the IDs 925354 and 872163 which, by contrast, showed the highest latex-responsive expression (Table 2B). The AA9 proteins are lytic polysaccharide monooxygenase (LPMO) commonly responsible for the cleavage of crystalline cellulose (Vaaje-Kolstad et al., 2010; Levasseur et al., 2013). They were the highly expressed candidates of *R. microporus* during the saprotrophic invasion on rubber wood (Oghenekaro et al., 2016b) as well as other saprotrophic fungi (Wymelenberg et al., 2011; Raffaello et al., 2014; MacDonald et al., 2011). On the other hand, the biotrophic pathogen *Ustilago maydis* lacks this class of LPMO protein (Figure 4), which aligns with previous evolutionary comparisons showing that *U. maydis* has lost its LPMO, GH6, and GH7 enzymes (Kohler et al., 2015). Furthermore, GH7 is the hydrolyzing enzyme cleaving the non-crystalline cellulose at the beta-1,4-glycosidic bond at the ends of the chain (Medie et al., 2012). GH7 was recognized as the family with the highest transcript abundance among other GH families during the analysis of *R. microporus*-rubber tree wood interaction. It was observed that a gene encoding GH7 protein (CL2079.Contig4) had a 71-fold increased expression and the two AA9 proteins (CL374.Contig3, CL374.Contig4) had more than 16-fold increased transcripts (Oghenekaro et al., 2016b). In response to latex, the expression of these two GHs was mostly suppressed, while some of them were induced (Table 2).

The aforementioned scenario illustrates the potential interaction, in which the WRD fungus largely utilizes several GHs as virulent factors for invading rubber trees and the rubber tree alleviates the rotting by the activity of its latex. When comparing among *R. microporus*-related fungal species in the order Hymenochaetales, the AA9 family (PF03443), GH7 proteins (PF00840), and the fungal cellulose binding domain (PF00734) contain more individual protein members in *R. microporus* and the powerful saprotrophic white rotter *Trichaptum abietinum* than in other species (Figure 4). The high candidate numbers of these proteins in WRD fungus and the rotter *T. abietinum* further support the potential contribution of two GH proteins to the evolutionary shift in the Hymenochaetales order, allowing these two fungi to cause rot symptoms on infected trees. Interestingly, several effector-related protein families of *R. microporus* contain a higher number of protein candidates compared to those in the white rot fungal pathogen *H. annosum* (Figure 4).

A high number of proteins belonging to the GH16 family (PF00722) were annotated in *R. microporus,* the bryophilous-associated fungus *Rickenella fibula*, as well as in the biotrophic pathogen *U. maydis* (Figure 4). The GH16 protein showed activity not only with the terrestrial polysaccharides but also with the marine polysaccharides (Viborg et al., 2019). As mentioned by Bradley et al. (2022), an example of GH16 from *Botrytis cinerea*, BcCrh1 protein, has been studied and proposed for its role in responsibility for infection and cell death induction on the host (Bi et al., 2021). Several GH16 proteins identified as apoplastic effectors of *R. microporus* were slightly triggered upon latex treatment (Supplementary Table S2). Notably, effector proteins containing Kre9/KNH-like N-terminal Ig-like domain (PF10342) were found only in *R. microporus*, *Rickenella fibula*, and *U. maydis*, but lacking in other representative Hymenochaetales species (Figure 4). They are mostly upregulated when challenged with the latex. The Kre9_KHN proteins were membrane-located proteins involved in the biosynthesis of fungal cell walls and the fruiting body formation (Szeto et al., 2007). The evidence to show the relevance of Kre9/KNH-like-domain-containing protein to the fungal pathogenicity is lacking, but their presence in *R. microporus* rather than most of other Hymenochaetales species together with the latex-responsive expression suggests the support of latex on the fungal growth as observed in Oghenekaro et al. (2020). This will facilitate the progressive increase of fungal mycelia and, consequently more rubber tree roots would be severely infected.

Cerato-platanin proteins (PF07249) are one of the conserved effector protein families in fungi (Gaderer et al., 2014; Luti et al., 2020), which were previously recognized for their role in the production of phytotoxin and triggering the systemic acquired resistance (SAR) and hypersensitive responses (HR) in plants (Frias et al., 2014; Frias et al., 2011). The proteins in this family have been suggested as crucial effectors utilized by fungal pathogens (Vasina et al., 2016; Pan et al., 2018; Zhang et al., 2020; Khairi et al., 2022). In the necrotrophic fungus *Sclerotinia sclerotiorum*, its cerato-platanin SsCP1 protein interacts with the pathogenesis-related protein 1 (PR-1) of *Nicotiana benthamiana* and contributes to its virulence (Yang et al., 2018). In *R. microporus*, Maiden et al. (2024) reported the stable expression of a gene encoding RmCP, a cerato-platanin homolog, during the infection into rubber trees. The analysis here identified 7 cerato-platanins (Table 1), which is the highest number compared to the number of cerato-platanins identified in other representative species in Hymenochaetales (Figure 4). Overall, the *R. microporus* effectors belonging to Cerato-platanin were upregulated against latex treatment, except for the slightly lowered ID 137475 (Supplementary Table S2).

Moreover, some of the identified unknown effectors of *R. microporus* that showed a high level of differential expression response to the rubber tree latex (Table 2B) were predicted for their tertiary structures by The I-TASSER server (Table 3; Figure 3). These key unknown effectors are proposed here to be the structural relatives of known proteins, noted as the E3 ubiquitin ligase-like protein (ID 882769), spherulin-like protein (ID 872712), aminopeptidase-like protein (ID 942071), and thaumatin-like protein (ID 941140).

Previously, E3 ubiquitin ligase activity was mentioned to be used by fungal pathogens to interfere with the proteasome of host plants and facilitate pathogenicity (Duplan and Rivas, 2014). The ubiquitination is a key process in regulating the secretion of fungal effectors, such as in the rice blast *Magnaporthe oryzae* (Wang et al., 2022). The predicted structure of the highly upregulated effector ID 882769 showed a similarity toward the E3 ubiquitin ligase (Figure 3E). Moreover, the spherulin 3A protein from the slime mold *Physarum polycephalum* was the template and the structural-related protein to the effector ID 872712 (Table 3; Figure 3F). Though the spherulin protein was also identified in the grape canker fungus *Botryosphaeria parva* (UniProt ID: R1EXI5), there is limited information on how spherulin proteins participate in the pathogenicity of plant fungal pathogens. However, spherulin is a starvation-related protein in fungi that exhibits the conserved H[I/T]HPRATEI to the germin-like proteins in plants. They are both considered members of cupin superfamily proteins (Dunwell et al., 2004). Another identified *R. microporus* effector containing RxLR motif (ID 888205) also belongs to the cupin family, although its expression was slightly lower. Therefore, the relatedness of the spherulin-like effector from *R. microporus* to the fungal virulence may resemble the pathogenicity mediated by a cupin-domain-containing effector named FoCupin1 from *Fusarium oxysporum* f. sp. *cubense* (Yan et al., 2022). Further functional investigation of these effectors regarding the effects of rubber tree latex on the fungal proteasome, secretome, as well as the protein interaction to the host immune merits to be investigated.

Thaumatin (PF00314) is well-known as an antifungal protein in plants in the PR-5 family (de Jesus-Pires et al., 2020). Because of the antifungal activity or cleaving of the beta-1,3-glucanase, the thaumatin-like proteins in *R. microporus* effector identified in this study (Supplementary Tables S2, S3) may not likely contribute to the pathogenicity of the WRD. It is most likely to contribute to the mycoparasitic activity in competing with other fungal organisms. In fungi, a thaumatin-like protein (TLP) was recently identified in *Rhizoctonia solani* and is being suggested as an elicitor protein triggering the defense of maize plants (Liu et al., 2024). Although a thaumatin-like protein was predicted as one of the upregulated *R. microporus* effectors, another thaumatin (ID 862870) was the most downregulated effector (Table 2A). The compensation of thaumatin expression in *R. microporus* upon latex treatment was also documented (Supplementary Table S2).

## 5 Conclusion

**Figure 5:**
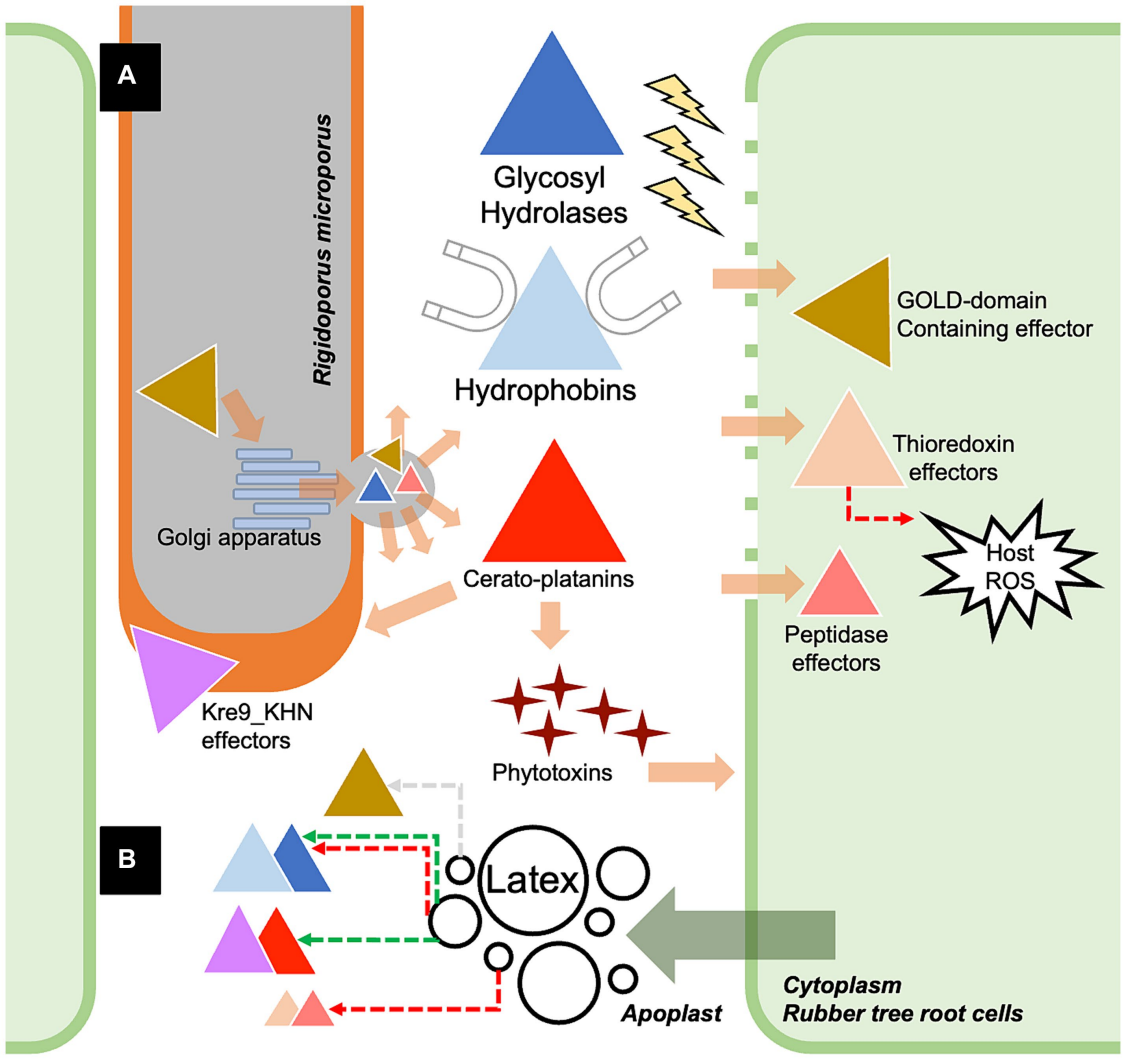
The proposed scenario showing the partial molecular pathogenicity of white root rot disease mediated by the identified effectors of *Rigidoporus microporus* fungus in this study **(A)** and the effects of rubber tree latex on the expression of some effectors **(B)**. Briefly, *R. microporus* exhibits GOLD-domain-containing effector proteins that may facilitate the secretion function via the Golgi apparatus, further promoting the release of fungal effectors toward the rubber tree cells. There are also several classes of glycoside hydrolases (GHs) proteins that are the apoplastic effectors that cleave the cell wall components of rubber trees. The hydrophobins are the common apoplastic effectors of *R. microprus* that also destroy the host cell wall and enable the attachment of fungus to the rubber tree’s cell surface. Furthermore, the hyphal-enhancing and phytotoxins-producing cerato-platanin proteins are another group of apoplastic effectors of *R. microporus*. GOLD-domain-containing effectors, thioredoxin effectors, and peptidase effectors are proposed as crucial cytoplasmic effectors of *R. microporus*, which interfere with the molecular defense of rubber trees. Although the latex released by the rubber tree showed suppressive activity against these fungal effectors, some of them could be induced by the latex based on the experiment by Oghenekaro et al. (2020). The fungal effectors from *R. microporus* are illustrated as triangles with different colors corresponding to each of the containing protein domains. The functional locations of effectors were demonstrated based on the prediction in Supplementary Table S2. The colors of dashed arrows indicate the downregulation (red) or upregulation (green) of effectors or the plant defense.

In conclusion, the dataset of putative secreted effectors from the white root rot fungus *Rigidoporus microporus* was explored for the first time based on the characteristics predicted by the combination of Signal P and Effector P servers, as well as the presence of fungal-conserved effector motifs. The cytoplasmic effectors and apoplastic effectors of *R. microporus* were categorized despite several of them being unknown effectors. Most of the known effectors belong to hydrophobins and GH, which facilitate invasive growth and the degradation of rubber tree cell walls. The dataset of the response toward the latex application was integrated and underlined the crucial groups of effectors which may be utilized by *R. microporus* to cause the WRD in rubber trees (Figure 5). Taken together, the *in silico* identification of effectors opens up the opportunity for experimental validation of the several prioritized effector candidates. It would also benefit the development of novel strategies for managing the WRD of rubber trees in future.

## Data Availability

The datasets presented in this study can be found in online repositories. The names of the repository/repositories and accession number(s) can be found in the article/Supplementary material.

## Footnotes

^1^https://www.cabidigitallibrary.org/doi/10.1079/cabicompendium.47610

^2^https://genome.jgi.doe.gov/portal/Rigmic1/Rigmic1.download.html

^3^https://services.healthtech.dtu.dk/services/SignalP-6.0/

^4^https://effectorp.csiro.au/

^5^https://meme-suite.org/meme/tools/fimo

^6^https://mycocosm.jgi.doe.gov/Rigmic1/Rigmic1.home.html

^7^https://www.ebi.ac.uk/Tools/hmmer/search/hmmscan

^8^https://www.ncbi.nlm.nih.gov/bioproject/?term=497786

^9^https://zhanggroup.org/D-I-TASSER/
